# Mice with humanized FXR ligand-binding domain display distinct metabolic responses upon pharmacological FXR stimulation

**DOI:** 10.1016/j.jlr.2026.101088

**Published:** 2026-06-25

**Authors:** Jinxiao Li, Hilde D. de Vries, Kirill Ustyantsev, Milaine V. Hovingh, Niels L. Mulder, Rick Havinga, Nicolette Huijkman, Sarah Falcone, Krisztina de Bruyn, Ellen Weersing, Niels J. Kloosterhuis, Marieke Smit, Eugene Berezikov, Bart van de Sluis, Henkjan J. Verkade, Folkert Kuipers, Jan Freark de Boer

**Affiliations:** 1 Department of Pediatrics, University of Groningen, University Medical Center Groningen (UMCG), Groningen, The Netherlands; 2 European Research Institute for the Biology of Ageing (ERIBA), University of Groningen, University Medical Center Groningen (UMCG), Groningen, The Netherlands; 3 Department of Laboratory Medicine, University of Groningen, University Medical Center Groningen (UMCG), Groningen, The Netherlands

**Keywords:** humanized mouse model, nuclear receptor, cytochrome P450, bile acid metabolism, cholesterol metabolism, obeticholic acid, liver

## Abstract

Farnesoid-X-receptor (FXR), a bile acid (BA)-activated nuclear receptor, is a therapeutic target for cholestatic and metabolic liver diseases. However, species differences in BA metabolism and FXR signaling hamper translation from mice to humans. The human FXR ligand-binding domain (LBD) structurally differs from the murine LBD, potentially impacting pharmacological responses. Therefore, we generated mice with “humanized” FXR by replacing the murine LBD by the human LBD (FXR-hLBD) and assessed its impact on BA and cholesterol metabolism. Male and female FXR-hLBD mice on wild-type (WT) or *Cyp2c70*^−/−^ backgrounds were compared with FXR-mLBD controls under non-stimulated conditions. Additionally, WT mice expressing FXR-mLBD or FXR-hLBD received either vehicle or obeticholic acid (OCA; 40 mg/kg/day, p.o.) for 7 days. FXR humanization did not alter hepatic or intestinal FXR expression levels. Under basal conditions, physiological parameters, liver pathology markers, and hepatic transcriptomes were similar between FXR-hLBD and FXR-mLBD mice on a WT C57BL/6J background and in mice with a human-like BA composition (*Cyp2c70*^−/−^). OCA did, however, elicit markedly stronger transcriptional responses in FXR-hLBD mice, including more pronounced suppression of hepatic BA synthesis genes and differential regulation of BA transporters. Intriguingly, pathways involved in cell proliferation and fibrogenesis were induced in FXR-hLBD mice. Furthermore, OCA lowered plasma cholesterol to a greater extent in FXR-hLBD than FXR-mLBD mice, primarily due to a reduction in HDL-cholesterol. FXR-hLBD mice resemble FXR-mLBD controls under basal conditions but exhibit enhanced responses to FXR agonism by OCA. This model may improve preclinical evaluation of FXR-targeting drugs in a translation-relevant context.

Farnesoid X receptor (FXR/*NR1H4*), a member of the nuclear receptor superfamily, is a bile acid (BA)-activated transcription factor that is highly expressed in liver and intestine (1, 2). Due to differential promoter usage and alternative mRNA splicing, human as well as murine FXR have four isoforms (i.e., FXRα1-4) derived from a single *FXR* gene (2, 3, 4). These isoforms display tissue-specific expression patterns (3, 4) and appear to regulate metabolic functions in an isoform-dependent manner (5, 6, 7, 8). All FXR isoforms can bind to inverted repeat-1 (IR-1) response elements on the DNA, while FXRα2 and FXRα4 were also found to bind to everted repeat-2 (ER-2) response elements (9). When endogenous agonists, such as primary BA chenodeoxycholic acid (CDCA), bind to the ligand-binding domain (LBD) of FXR, a conformational change is induced that leads to the dissociation of corepressors and recruitment of coactivators. This, in turn, triggers the transcription of target genes. It was recently reporte008d that FXR can not only regulate gene expression as a heterodimer with the retinoid X receptor (RXR), but also as a monomer, via ER-2 motifs (2, 9). Importantly, the LBD is shared between all four FXR isoforms, suggesting isoforms will not be selectively activated by FXR ligands (1).

FXR functions as an intracellular BA sensor and regulates BA homeostasis (10). Activation of FXR induces small heterodimer partner (*SHP*/*NR0B2*) expression in the liver and subsequently reduces transcription of cholesterol 7α-hydroxylase (CYP7A1) (11), which catalyzes the rate-limiting step in BA synthesis. In the intestine, FXR mediates fibroblast growth factor 15/19 (*Fgf15/FGF19*) transcription, which also leads to inhibition of hepatic CYP7A1 following its binding to the FGF receptor 4 (FGFR4)/β-Klotho complex on hepatocytes (12). Additionally, FXR activation promotes transcription of the bile salt export pump (*BSEP*/*ABCB11*), while it limits hepatic BA uptake by repressing sodium taurocholate co-transporting polypeptide (*NTCP*/*SLC10A1*) expression (13). These effects serve to protect the liver from excessive BA exposure (14). Hence, pharmacological stimulation of FXR has emerged as a strategy for treating cholestatic liver diseases. Moreover, due to its essential role in modulating lipid metabolism, FXR is also acknowledged as a therapeutic target in metabolic dysfunction-associated steatotic liver disease (MASLD) and atherosclerotic cardiovascular disease (ASCVD) (15, 16).

A significant part of preclinical research aimed at developing novel therapeutics for the aforementioned diseases is carried out in mouse models. However, it is evident that species-specific differences in BA metabolism and signaling limit extrapolation of results from preclinical studies to the human situation. The identification of CYP2C70 as the enzyme responsible for muricholic acid (MCA) production (17, 18) and the subsequent development of *Cyp2c70*-deficient mice with a human-like BA composition have already provided important new insights (18, 19). In addition to the evident differences in BA pool composition between mice and humans, Cui *et al.* demonstrated that small variations in the amino acid sequence of the FXR-LBD between mice and humans considerably impact ligand-induced receptor activation in vitro. The human FXR-LBD was shown to exhibit a 10-fold greater affinity for the potent endogenous FXR agonist CDCA than the murine FXR-LBD. Moreover, the human FXR-LBD also displayed a 3-fold higher maximal response (20). Obeticholic acid (OCA/6-ECDCA/INT-747), a synthetic CDCA derivative (21), has been shown to potently activate human FXR (22, 23), yet multiple studies have described very modest transcriptional and metabolic effects upon OCA administration in mice, especially when OCA was provided via the diet rather than as a bolus given by oral gavage (24, 25, 26, 27, 28, 29).

To address the species differences in endogenous and/or pharmacological FXR activation, we generated a mouse model with “humanized” FXR in which the murine LBD is replaced by the human LBD (FXR-hLBD). Importantly, endogenous murine *Fxr* promoters as well as splice sites were retained to preserve all FXR isoforms in this model. In this study, we first characterized the phenotype of FXR-hLBD mice on a wild-type (WT) as well as on a *Cyp2c70*^−/−^ background under basal conditions. We found that male and female FXR-hLBD mice on both backgrounds were phenotypically indistinguishable from FXR-mLBD controls. Next, using the FXR agonist OCA, we investigated the impact of FXR-LBD humanization on hepatic metabolism upon pharmacological FXR activation. FXR-hLBD mice exhibited enhanced transcriptional and metabolic responsiveness to OCA compared to FXR-mLBD controls, illustrating that the interspecies FXR structural variation significantly impacts pharmacological responses in vivo.

## Materials and Methods

### Generation of FXR-hLBD mice

Whole-body FXR-hLBD mice were generated using CRISPR/Cas9 technology. The single-guide RNA (sgRNA; 5′-GTCATGTACAGATTCTCGTA-3′) targeting exon 8 of the murine *Fxr* gene, where the LBD starts, was synthesized by an in vitro transcription reaction using the EnGen® sgRNA Synthesis Kit (#E3322, New England Biolabs GmbH, Frankfurt am Main, Germany), according to the manufacturer’s instructions. The DNA repair template was ordered at Integrated DNA Technologies (IDT, Coralville, IA, USA), including homology arms, the complete coding sequence of the human *FXR-LBD*, and an SV40 polyadenylation signal downstream of the stop codon to ensure transcriptional termination. The DNA repair template sequence is provided in Supplemental Table S1.

Zygotes were harvested from mated female C57BL/6J mice and microinjected with a complex of 10 μg/μl Cas9 nuclease (#1081059, IDT), 121 ng/μl sgRNA, and 100 ng/μl DNA repair template. The zygotes were then transplanted into the ampulla via the infundibulum of pseudo-pregnant B6CBAF1/J females. The targeted region of the *Fxr* gene was sequenced in the offspring. Mosaic mice were crossed with WT (FXR-mLBD) C57BL/6J mice to generate heterozygous founders. Homozygous FXR-hLBD mice and their FXR-mLBD littermates were bred at the Central Animal Facility (University of Groningen, Groningen, the Netherlands).

### Genotyping

Genomic DNA was extracted from ear tissue using the lysis buffer (250 mM NaOH, 2 mM Na_2_EDTA) and amplified with two pairs of primers, including a universal forward primer 5′-AGGAAGTCTCAGGTCTATCTTGT-3′, a reverse primer specific to the murine LBD 5′-GAAAAACACCTAGCAAAAGAAATCC-3′, and a reverse primer specific to the human LBD 5′-TCAGCAAAGCAATCTGGTCTT-3’ (Supplemental Fig. S1A). PCR products were separated by 2% agarose gel electrophoresis and visualized using a gel imaging system (Bio-Rad). Genotypes were identified by product sizes corresponding to mLBD (587 bp) and hLBD (304 bp) (Supplemental Fig. S1B).

### Animals

Homozygous male and female FXR*-*mLBD and FXR-hLBD mice on a WT or *Cyp2c70*^−/−^ background (12–13 weeks old) were included in this study. FXR-KO mice, previously described by our group (30), were used as negative controls when indicated. All animals were housed individually in a temperature-controlled (20–22°C) facility with a 12-h light/dark cycle. Mice were fed a standard irradiated chow diet (Ssniff® 1554R/M-H maintenance diet) and had ad libitum access to food and drinking water. In a separate cohort, 8- to 11-week-old male FXR*-*mLBD and FXR-hLBD mice were treated once daily by oral gavage with either vehicle (0.5% sodium carboxymethyl cellulose; #21902, Sigma-Aldrich, St. Louis, MO) or OCA at a dose of 40 mg/kg body weight/day (dissolved in vehicle solution) for 7 days. Fecal samples were collected during the final 48 h of treatment. Mice received the last OCA bolus and were then fasted for 4 h prior to sacrifice by cervical dislocation after cardiac puncture under isoflurane anesthesia. Organs and tissues were excised and snap-frozen in liquid nitrogen. Blood was centrifuged at 8000 rpm for 10 min at 4°C and plasma was stored at −80°C until further analysis. Animal experiments were approved by the Animal Welfare Body of the University of Groningen and the Dutch Central Committee for Animal Experiments (No. AVD10500202115447), and procedures were performed in accordance with institutional and national guidelines and regulations.

### Quantitative RT-PCR

Total RNA was extracted from snap-frozen tissues using TRI-Reagent (#T9424, Sigma-Aldrich), according to the manufacturer's instructions. RNA concentrations were measured using a NanoDrop Microvolume Spectrophotometer (Thermo Fisher Scientific, Waltham, MA). For cDNA synthesis, RNA was reverse transcribed using Moloney Murine Leukemia Virus (M-MLV) Reverse Transcriptase (# 28025013, Invitrogen, Thermo Fisher Scientific) and Random Nonamers (#R7647, Sigma-Aldrich) according to the manufacturer’s protocol. Quantitative RT-PCR was performed on a QuantStudio 7 System (Applied Biosystems) using SYBR green mastermix (#A25743, Thermo Fisher Scientific). Primer sequences are listed in Table S2. Gene expression was normalized to *Cyclophilin G* (*Ppig*) as a housekeeping gene.

### RNA library preparation and sequencing

The RNA-Seq libraries were prepared at iPSomics (Groningen, the Netherlands) according to the Smart-3SEQ protocol (31) using 100 ng of total RNA per sample as input. The libraries were pooled and sequenced on Illumina NovaSeq X platforms using a 150 bp paired-end sequencing strategy at Novogene Europe (Cambridge, UK). Sequence reads were mapped to the mouse reference genome (GRCm38/mm10) and gene counts were calculated with featureCounts (32), allowing for multiple mapping. Differential gene expression was determined using DESeq2 (v1.46.0) package (33) with False Discovery Rate (FDR) correction applied using the Benjamini-Hochberg method. Gene Set Enrichment Analysis (GSEA) was performed using clusterProfiler (v4.14.6) package (34).

### Nanopore long-read cDNA sequencing

Libraries were prepared with cDNA-PCR Sequencing V14-Barcoding (SQK-PCB114.24) using 2 μg of total RNA input. The PCR-amplified barcoded cDNA libraries were pooled equimolarly, loaded into FLO-PRO114M flow cell and sequenced on a PromethION 2 Solo (P2 Solo) device (Oxford Nanopore Technologies) for 72 h. De novo isoform reconstruction and quantification were performed using isON_pipeline.sh from the isONform software package v.0.3.6 (35).

### Western blotting

Frozen liver tissues were homogenized (10%, w/w) in Nonidet P40 (NP40) buffer containing 0.1% NP40, 0.4 M NaCl, 10 mM Tris-HCl (pH 8.0), and 1 mM EDTA, supplemented with protease inhibitors (cOmplete, #11836145001, Roche, Basel, Switzerland). Total protein levels were quantified using the Pierce BCA protein assay kit (#23225, Thermo Fisher Scientific). Equal amounts of proteins were loaded and separated by SDS-PAGE gel electrophoresis using 4%–15% Mini-PROTEAN® TGX™ Precast Protein Gels (#4561084, Bio-Rad) and transferred to PVDF membranes (0.2 μm; #1704157, Bio-Rad) using a semi-dry Trans-Blot Turbo Transfer System (Bio-Rad). Membranes were blocked in 5% bovine serum albumin (BSA) in Tris-buffered saline solution with Tween 20 (TBST) for 1 h and then incubated with primary antibodies against FXR (1:1000; #PP-A9033A-00, Perseus Proteomics, Tokyo, Japan) and HSP90 (1:1000; #4874, Cell Signaling Technology) overnight at 4°C. Membranes were then washed with TBST and incubated with HRP-conjugated secondary antibody goat anti-mouse IgG/HRP (1:2000; #1706516, Bio-Rad) or goat anti-rabbit IgG/HRP (1:10,000; #P0448, DAKO) for 1 h at room temperature. Immune complexes were visualized using SuperSignal West Femto substrate (#34578, Thermo Fisher Scientific). Membranes were imaged using a ChemiDoc MP Imaging System (Bio-Rad) and quantified using Image Lab software (v5.2.1, Bio-Rad).

### Measurement of plasma lipids and liver function markers

Plasma transaminases and bilirubin were determined using a Cobas 6000 analyzer with standard reagents (Roche Diagnostics). Plasma total cholesterol levels were measured using the commercial kit (#113009910026, DiaSys Diagnostic Systems GmbH, Holzheim, Germany) and a Cholesterol Standard (#113003010030, DiaSys Diagnostic Systems). Lipoprotein profiles were analyzed using fast protein liquid chromatography (FPLC) as described before (36).

### Histological analysis

Liver tissues were fixed in 4% (w/v) formalin, embedded in paraffin, and sectioned at 4 μm. Hematoxylin and eosin (H&E) and Sirius red/fast green (SrFg) stainings were performed using standard protocols. Images were acquired using a Hamamatsu NanoZoomer (Hamamatsu Photonics, Almere, the Netherlands). Stained areas were quantified using ImageJ (v1.54d, National Institutes of Health, Bethesda, MD).

### Bile acid measurements

Deuterium-labeled internal standards, dissolved in MeOH, were added to plasma and bile samples. Following centrifugation, the supernatants were transferred to a 96-well plate and fluid was evaporated. The samples were then redissolved in 50% MeOH. Fecal samples were thoroughly dried, ground, and weighed. Approximately 30 mg was then dissolved in a 1:3 mixture of 0.5 M NaOH and MeOH and incubated for 3 h at 80°C. After addition of deuterium-labeled internal standards, BAs were extracted by solid-phase extraction using Oasis HLB 96-well plates (Waters). After elution, the liquid was evaporated and BAs were redissolved in 50% MeOH.

Chromatographic separation of the analytes was performed with a Waters 2D ultra-high performance liquid chromatography (UPLC) system operated in one-dimensional mode, using a reversed-phase Waters Acquity UPLC BEH C18 column (2.1 × 100 mm, 1.7 μm). Column temperature was maintained at 40°C. Analytes were eluted using 10 mM ammonium acetate with 20% acetonitrile (mobile phase A) and 10 mM ammonium acetate with 80% acetonitrile (mobile phase B), at a flow rate of 0.4 ml/min. Initial conditions were 96:4 (v/v) mobile phase A:mobile phase B, followed by an exponential increase of mobile phase B to 5% during 5 min, and 50% during 8 min. Thereafter, mobile phase B was increased to 95% in 1 min, and was kept constant for another minute. The mobile phases were then returned to the initial 96:4 ratio and kept constant for 1 min, giving a total run time of 16 min. Mass spectrometric detection of analytes was performed in negative ionization mode (ESI-) with a Waters Xevo TQ-S micro triple quadrupole mass spectrometer.

### Statistical analysis

Data are presented as bar plots or Tukey box-and-whisker plots using GraphPad Prism (v9.0). Mendelian ratios were analyzed using Pearson’s Chi-square test. Differences between two groups were analyzed using Mann-Whitney *U*-test, while comparisons among multiple groups were performed by Kruskal-Wallis H-test, followed by Conover post hoc analysis (BrightStat (37)). A *P*-value < 0.05 was considered statistically significant.

## Results

### Generation of a mouse model with a humanized FXR ligand-binding domain

**Fig. 1:**
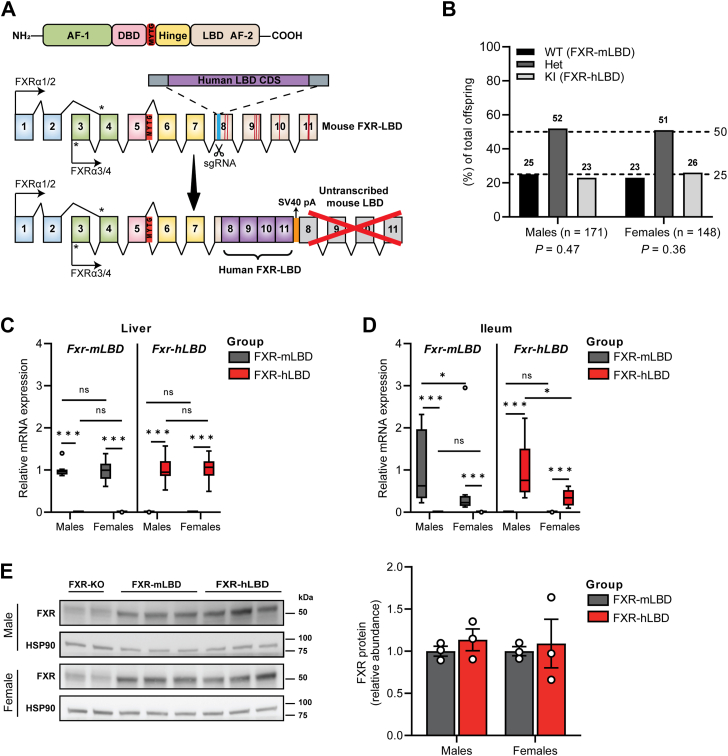
FXR-hLBD mice display normal Mendelian ratios and express humanized Fxr in liver and ileum. A: Schematic representation of the CRISPR/Cas9 strategy to replace the murine FXR ligand-binding domain (LBD; exons 8–11, beige) with the human FXR-LBD (purple), followed by an SV40 polyadenylation signal (orange) for transcriptional termination. Asterisks indicate the translational start sites (ATG) of the isoforms. Red lines indicate positions of amino acid differences between the murine and human LBD. (B) Genotype distribution of pups from FXR-mLBD/hLBD heterozygous breeding pairs. Numbers above bars indicate observed percentages. Dashed lines indicate the expected Mendelian distribution. N = 319 pups, including 171 males and 148 females. Mendelian ratios were tested using Pearson’s Chi-square test. C–D: Relative mRNA expression of *Fxr-mLBD* and *Fxr-hLBD* in liver (C) and ileum (D), quantified by RT-qPCR. N = 8–9 mice/group. E: Immunoblotting analysis (left) of liver lysates for FXR and HSP90 (loading control). FXR-KO mouse samples were included as negative controls. Quantified band intensities were normalized to HSP90 protein (n = 3 mice/group; right), showing mean ± SEM. Data are presented as a bar plot and Tukey box-and-whisker plots. ns, not significant, ∗∗∗*P* < 0.001 by Kruskal-Wallis H test, followed by Conover post hoc comparisons. AF-1, activation function-1; AF-2, activation function-2; CDS, coding sequence; DBD, DNA-binding domain; Het, heterozygous; KI, knock-in; LBD, ligand-binding domain; WT, wild type.

Although the FXR-LBD is highly conserved between humans and mice, key amino acid differences (Supplemental Fig. S2) have been reported to affect receptor sensitivity to BA activation (20). To investigate potential consequences of these species-specific differences in vivo, we generated mice with a humanized FXR by replacing the murine LBD with the human LBD using CRISPR/Cas9 technology, while retaining all other regions of murine FXR (Fig. 1A; see Materials and Methods for details). FXR-hLBD mice of both sexes were born at the expected Mendelian ratios (Fig. 1B), appeared healthy, and showed no apparent abnormalities during development and adulthood.

Quantitative RT-PCR analysis demonstrated mRNA expression of *Fxr-hLBD* in the liver and ileum of FXR-hLBD mice, with a complete absence of *Fxr-mLBD* expression in these animals. Conversely, only *Fxr-mLBD* was detected in WT controls (Fig. 1C, D). Hepatic *Fxr* transcript levels were comparable between sexes, whereas ileal expression was lower in females as compared to males in FXR-hLBD mice as well as in WT controls. Immunoblotting demonstrated similar hepatic FXR protein levels in FXR-hLBD and WT controls of both sexes (Fig. 1E), indicating that LBD humanization did not affect overall receptor expression in the liver.

### Preservation of Four FXRα isoforms with humanized LBD in FXR-hLBD mice

**Fig. 2:**
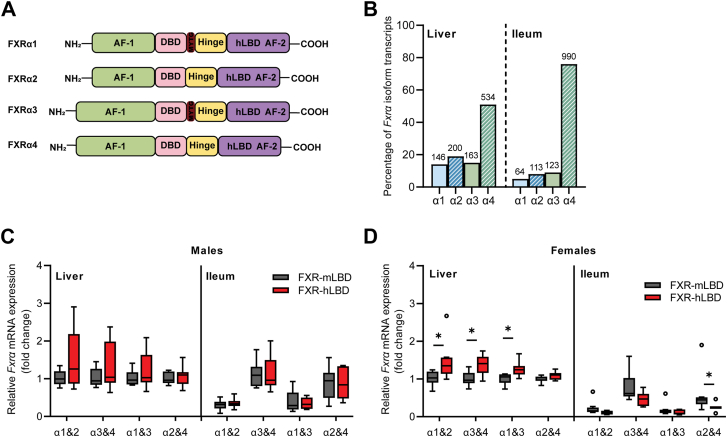
*Fxrα* isoforms are preserved in FXR-hLBD mice. A: Schematic illustration of the four FXRα isoforms (FXRα1-4) in FXR-hLBD mice. B: Percentage distribution of *Fxrα* isoform transcripts in liver and ileum. Numbers above the bars indicate the total number of reads for each isoform, as determined by Nanopore long-read cDNA sequencing from one male FXR-hLBD mouse. C–D: Relative mRNA expression of *Fxrα* isoforms in liver and ileum of males (C) and females (D), quantified by RT-qPCR (n = 8 mice/group). Expression of *Fxrα1&2* in FXR-mLBD livers was used as the reference group. Asterisks represent significant differences between FXR-mLBD and FXR-hLBD groups (∗*P* < 0.05), using Mann-Whitney U test. Data are presented as a bar plot and Tukey box-and-whisker plots. AF-1, activation function-1; AF-2, activation function-2; DBD, DNA-binding domain; LBD, ligand-binding domain.

Nanopore long-read cDNA sequencing confirmed the presence of all four mouse *Fxrα* isoforms with the humanized LBD (Fig. 2A), in the liver and ileum of FXR-hLBD mice (Fig. 2B), with *Fxrα4* being the predominant form in both tissues (51.2% liver; 76.7% ileum). The expression levels of *Fxrα1-3* were moderate and comparable in the liver, whereas the *Fxrα1* transcript was lowest in the ileum.

We next evaluated the relative expression of isoforms in FXR-hLBD mice as compared to WT controls using isoform-specific primers. No significant differences in expression of *Fxr* isoforms were observed between genotypes in males (Fig. 2C). Despite the aforementioned identical hepatic FXR protein concentrations (Fig. 1E), hepatic transcript levels of *Fxr*-isoforms, except *Fxrα2&4*, were higher in female FXR-hLBD mice compared to controls, whereas the ileal expression of *Fxrα2&4* was lower in FXR-hLBD mice (Fig. 2D). In the ileum of both sexes, expression of *Fxrα3&4* was higher than *Fxrα1&2*, consistent with data reported previously (4). Isoforms with an MYTG insert (*Fxrα1&3*) were expressed at lower levels than those without an insert (*Fxrα2&4*) in the ileum (Fig. 2C, D). Collectively, these data demonstrate that all four *Fxrα* isoforms are preserved in FXR-hLBD mice and that isoform expression patterns were largely similar in FXR-hLBD mice and FXR-mLBD controls.

### Humanization of the FXR-LBD does not induce phenotypic changes or impact hepatic gene expression patterns in mice under basal conditions

FXR humanization in mice did not impact food intake, body weight, organ weights, or body composition (i.e., fat and lean mass) when compared to their respective controls expressing endogenous FXR (Table 1). Importantly, liver enzymes and total bilirubin levels in plasma did not differ between genotypes, with only modestly decreased aspartate aminotransferase (AST) in female FXR-hLBD mice (Table 1). Histological examination showed no major differences in liver morphology between the groups (Supplemental Fig. S3A), indicating absence of liver pathology in FXR-hLBD mice. Furthermore, parameters of BA metabolism remained unaffected. Biliary and plasma BA compositions were highly comparable between genotypes, with no differences in the hydrophobicity index (HI) of biliary BAs (Supplemental Fig. S3B–D) and no differences in plasma BA levels (Table 1).

**Table 1:** Characteristics of young-adult FXR-mLBD and FXR-hLBD mice

Parameters	Males		Females	
	FXR-mLBD N = 7-8	FXR-hLBD N = 8	FXR-mLBD N = 7-8	FXR-hLBD N = 9
Food intake (g/day)	4.7 [4.6–4.8]	4.7 [4.6–4.7]	4.5 [4.3–4.6]	4.4 [4.2–4.6]
Body weight (g)	26.9 [26.0–27.5]	27.4 [25.3–28.1]	21.3 [20.8–22.1]	21.8 [21.0–22.3]
Liver weight (% of BW)	5.18 [4.97–5.28]	5.25 [4.92–5.42]	4.97 [4.85–5.17]	4.85 [4.79–4.99]
Spleen weight (% of BW)	0.25 [0.24–0.26]	0.25 [0.24–0.27]	0.35 [0.34–0.37]	0.35 [0.33–0.35]
Fat mass (g)	3.0 [2.5–3.3]	2.8 [2.4–3.4]	1.6 [1.5–2.1]	2.4 [1.7–2.7]
Lean mass (g)	20.5 [19.6–21.2]	20.3 [19.7–21.6]	17.0 [16.8–18.0]	17.0 [16.9–17.7]
Plasma				
AST (U/L)	70 [60–84]	78 [69–91]	110 [93–115]	75 [70–90]^a^
ALT (U/L)	25 [20–38]	40 [34–51]	40 [34–70]	30 [25–35]
ALP (U/L)	83 [75–86]	83 [79–88]	118 [110–124]	125 [115–130]
Total bilirubin (μmol/L)	<1	1 [0–2]	2 [1–2]	1 [1–2]
Plasma bile acids (μmol/L)	2.19 [1.77–3.65]	3.15 [1.85–33.17]	20.89 [10.59–36.44]	8.82 [7.12–13.68]

Note: Values are presented as median [interquartile range].

ALP, alkaline phosphatase; ALT, alanine aminotransferase; AST, aspartate aminotransferase; BW, body weight; LBD, ligand-binding domain.

a *P* < 0.05 for FXR-hLBD versus FXR-mLBD by Mann-Whitney U test.

FXR maintains metabolic homeostasis through regulating a large number of target genes (1). To evaluate whether FXR humanization impacts hepatic gene expression patterns under basal conditions, RNA sequencing was performed. Hepatic transcriptome profiles were highly similar between genotypes, with no differences in *Fxr*-targeted signaling in either sex (Supplemental Fig. S3E, F). These data demonstrate that, on a C57BL/6J background, FXR-hLBD mice exhibit normal development and hepatic gene expression patterns similar to FXR-mLBD mice under basal, non-stimulated conditions.

Because the murine BA pool constitute ∼50% of hydrophilic MCAs (see Supplemental Fig. S3B, D) with described FXR antagonistic actions (38), we cross-bred the FXR-hLBD into *Cyp2c70*^−/−^ mice with a human-like, CDCA-rich BA pool (36). Analysis of plasma BA concentrations and composition revealed no significant differences between *Cyp2c70*^−/−^ mice with either FXR-hLBD or FXR-mLBD and confirmed the very high proportion of CDCA, particularly in females (Supplemental Fig. S4A, B). Yet, neither male nor female *Cyp2c70*^−/−^ mice showed changes in phenotypic characteristics due to the presence of FXR-hLBD (Supplemental Fig. S4C). Plasma liver enzyme levels (known to be elevated in female *Cyp2c70*^−/−^ mice (36)) were not altered (Supplemental Fig. S4D). Hepatic and ileal expression of established FXR target genes (i.e., *Shp, Lcn13*, *Fgf15*, and *Ibabp*) did not differ between genotypes. Only hepatic *Abcb11* (*Bsep*) expression was significantly upregulated in female *Cyp2c70*^−/−^/FXR-hLBD mice, but not in males (Supplemental Fig. S4E). These results indicate that, under basal conditions, humanization of the FXR-LBD does not impact BA homeostasis and liver function in mice.

### FXR-hLBD mice exhibit enhanced transcriptional responses of genes involved in bile acid metabolism upon OCA administration

**Fig. 3:**
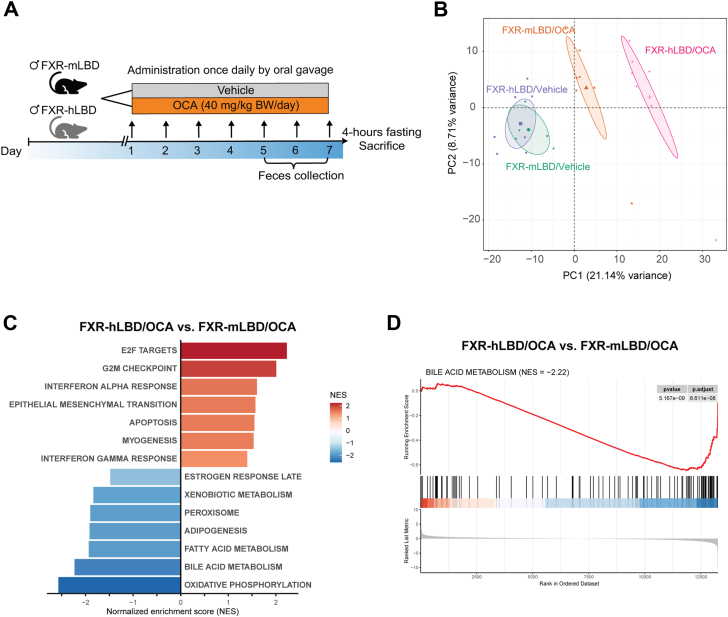
FXR-hLBD mice display a distinct transcriptome profile with marked suppression of Hallmark bile acid metabolism gene set upon OCA administration. A: Experimental timeline. Male FXR-mLBD and FXR-hLBD mice were treated via oral gavage once daily with vehicle (0.5% sodium carboxymethyl cellulose) or OCA (40 mg/kg/day in vehicle) for 7 days (n = 4–8 mice/group). B: Principal component analysis (PCA) of hepatic RNA-seq profiles. Each dot represents a biological replicate. C: Top upregulated and downregulated Hallmark gene sets in FXR-hLBD/OCA versus FXR-mLBD/OCA mice (FDR < 0.05). DESeq2 output was ranked by the Wald statistic, prior to GSEA. D: GSEA plot of the Hallmark bile acid metabolism gene set in FXR-hLBD/OCA versus FXR-mLBD/OCA mice. LBD, ligand-binding domain; OCA, obeticholic acid; PC, principal component.

Next, to evaluate the impact of FXR-LBD humanization on hepatic metabolism upon pharmacological FXR activation, FXR-hLBD and FXR-mLBD mice were treated with either vehicle or OCA (40 mg/kg/day) via oral gavage for 7 days (Fig. 3A) and RNA sequencing was performed on livers of these animals. Principal component analysis (PCA) was then performed to evaluate overall changes in hepatic gene expression patterns. The vehicle-treated groups of FXR-mLBD and FXR-hLBD overlapped, confirming that the humanization of FXR did not affect hepatic gene expression under basal conditions. OCA administration induced a clear separation from vehicle groups in both FXR-mLBD and FXR-hLBD mice, mainly along PC1 (Fig. 3B). Interestingly, FXR-hLBD mice showed a markedly more pronounced transcriptional shift than FXR-mLBD mice upon OCA treatment. Next, GSEA was performed to identify the most up-/downregulated Hallmark gene sets (39) between groups (Fig. 3C and Supplemental Fig. S5A, B). Consistent with the well-established role of FXR activation in inhibiting BA synthesis (1), BA metabolism was identified as the second most downregulated gene sets in FXR-hLBD livers relative to FXR-mLBD controls upon OCA administration (NES = −2.22; *P*_*adj*_ < 10^−7^; Fig. 3D).

**Fig. 4:**
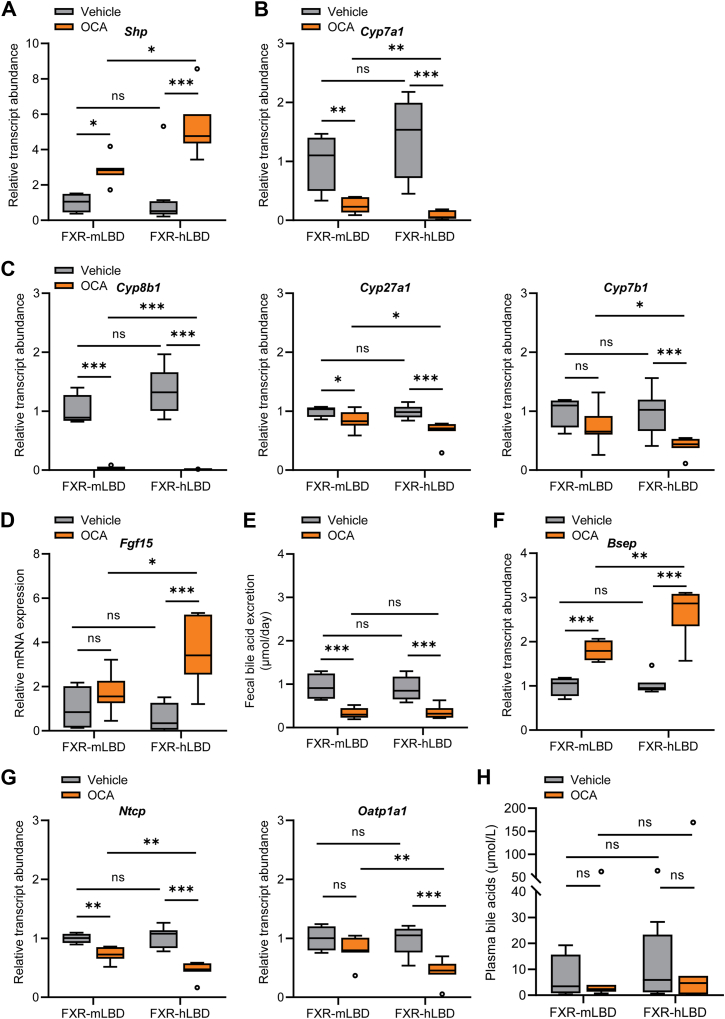
OCA elicits more pronounced transcriptional responses in FXR-hLBD mice than in FXR-mLBD controls. A–C: Hepatic transcript abundance of the *Fxr*-target gene *Shp/Nr0b2* (A), the gene encoding the rate-limiting BA synthesis enzyme *Cyp7a1* (B), and other major BA synthesis genes (*Cyp8b1*, *Cyp27a1*, *Cyp7b1*; C), calculated from normalized RNA-seq data. D: Ileal mRNA expression of the intestinal *Fxr*-target gene *Fgf15*, quantified by RT-qPCR. E: Fecal BA excretion. (F–G) Hepatic transcript abundance of (F) BA efflux transporter *Bsep*/*Abcb11* and (G) BA reuptake transporters *Ntcp*/*Slc10a1* and *Oatp1a1*/*Slco1a1*, calculated from normalized RNA-seq data. H: Plasma BA levels. Data are presented as Tukey box-and-whisker plots. ns, not significant, ∗*P* < 0.05, ∗∗*P* < 0.01, ∗∗∗*P* < 0.001 by Kruskal-Wallis H test, followed by Conover post hoc comparisons. BA, bile acid; LBD, ligand-binding domain; OCA, obeticholic acid.

Hepatic *Shp* expression was significantly upregulated by OCA in both genotypes, with a considerably stronger increase in FXR-hLBD mice than in FXR-mLBD (Fig. 4A). Consistently, mRNA expressions of *Cyp7a1*, *Cyp8b1*, and *Cyp27a1*, encoding key BA synthesis enzymes, was suppressed by OCA in both groups, with the strongest effects observed in FXR-hLBD mice. OCA-induced downregulation of *Cyp7b1* was significant in FXR-hLBD livers only (Fig. 4B, C). In the ileum, FXR-hLBD mice, but not FXR-mLBD controls, showed a marked increase in *Fgf15* mRNA levels in response to OCA treatment (*P* < 0.001; Fig. 4D). In line with the substantially reduced expression of *Cyp7a1*, encoding cholesterol 7α-hydroxylase, which is the rate-limiting enzyme in BA synthesis, OCA significantly reduced fecal BA excretion in both genotypes (Fig. 4E).

Hepatic expression of *Bsep*, which mediates canalicular BA efflux, was highly upregulated by OCA in both genotypes. Considerably stronger increases were, however, observed in FXR-hLBD mice (*P* < 0.01 vs. OCA-treated FXR-mLBD mice; Fig. 4F). Conversely, expression levels of BA uptake transporters *Ntcp* (*Slc10a1*) and organic anion transporting polypeptides (*Oatp1a1/Slco1a1*) were reduced more strongly in FXR-hLBD than in FXR-mLBD livers upon OCA treatment (Fig. 4G). Despite these transcriptional changes, circulating plasma BA levels remained similar in all groups (Fig. 4H).

**Fig. 5:**
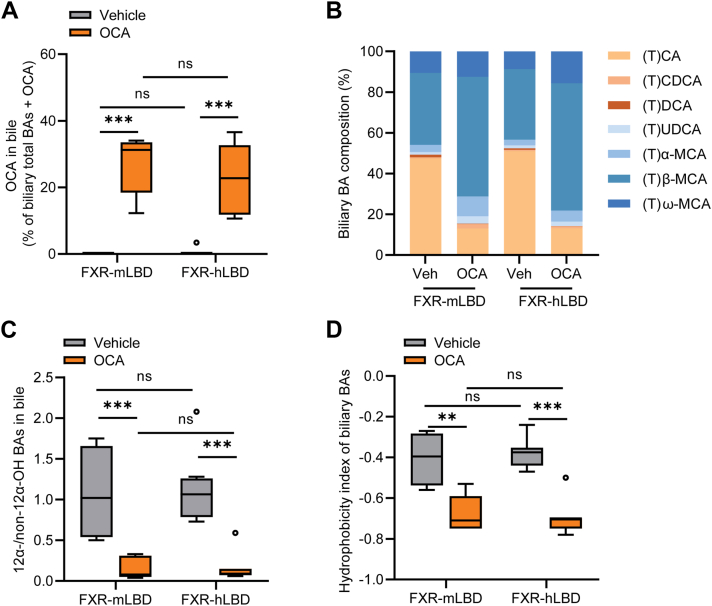
OCA reduces 12α-hydroxylated bile acids and induces a more hydrophilic biliary bile acid composition in both FXR-mLBD and FXR-hLBD mice. A: OCA in bile expressed as a percentage of the sum of biliary total BAs and OCA. (B: BA composition in bile. C: 12α-/non-12α-OH BA ratios in bile. D: Hydrophobicity index of biliary BAs. N = 4–8 mice/group. Data are presented as Tukey box-and-whisker plots and a stacked bar plot. ns, not significant, ∗*P* < 0.05, ∗∗*P* < 0.01, ∗∗∗*P* < 0.001 by Kruskal-Wallis H test, followed by Conover post hoc comparisons. BA, bile acid; LBD, ligand-binding domain; OCA, obeticholic acid; Veh, vehicle.

We next asked whether LBD humanization altered BA composition in response to OCA. The relative abundance of OCA in bile was similar between genotypes (Fig. 5A). In line with reduced hepatic sterol 12α-hydroxylase (*Cyp8b1)* expression, the relative abundance of 12α-hydroxylated cholic acid (CA) and deoxycholic acid (DCA) was markedly reduced in OCA-treated animals (Fig. 5B and Supplemental Fig. S6A), while non-12α-hydroxylated BAs, i.e., CDCA, MCAs and ursodeoxycholic acid (UDCA), became proportionally more abundant (Fig. 5B, C). Because MCAs are highly hydrophilic, these compositional changes translated into a more hydrophilic biliary BA composition, as indicated by a reduced hydrophobicity index in OCA-treated mice (Fig. 5D). These data are consistent with our previous observations in C57BL/6J mice (25). Although OCA treatment considerably impacted BA composition compared to vehicle, no major differences in BA profiles were observed between genotypes, indicating that LBD humanization did not significantly affect changes in biliary BA composition induced by OCA administration.

### OCA more effectively decreases plasma cholesterol levels in FXR-hLBD mice

**Fig. 6:**
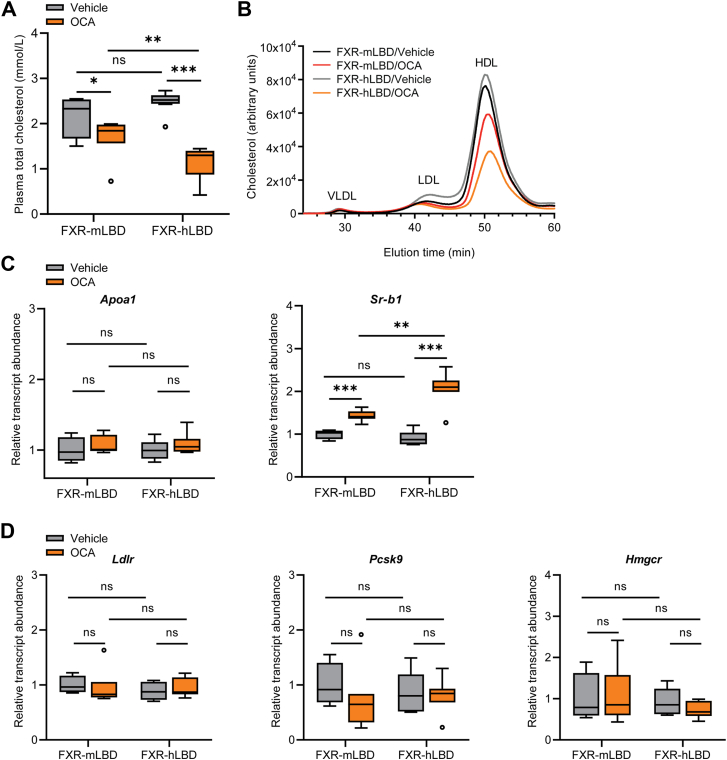
OCA more effectively decreases plasma cholesterol levels in FXR-hLBD mice. A: Plasma total cholesterol levels (n = 4–8 mice/group). B: Cholesterol distribution after lipoprotein fractionation of pooled plasma samples within each experimental group. C–D: Hepatic transcript abundance of (C) *Apoa1*, *Sr-b1*/*Scarb1*, and (D) *Ldlr*, *Pcsk9*, *Hmgcr*, calculated from normalized RNA-seq data. Data are presented as Tukey box-and-whisker plots or line plot. ns, not significant, ∗*P* < 0.05, ∗∗*P* < 0.01, ∗∗∗*P* < 0.001 by Kruskal-Wallis H test, followed by Conover post hoc comparisons. HDL, high-density lipoprotein; LBD, ligand-binding domain; LDL, low-density lipoprotein; OCA, obeticholic acid; VLDL, very low-density lipoprotein.

Pharmacological FXR activation has been associated with alterations in the lipoprotein profile in healthy individuals (40), and in patients with primary biliary cholangitis (PBC) (22, 23, 41) or non-alcoholic steatohepatitis (42, 43). We therefore examined whether FXR-LBD humanization impacts cholesterol metabolism upon OCA administration. Plasma total cholesterol levels were reduced by 48% in FXR-hLBD mice compared to only 26% in FXR-mLBD controls (*P* < 0.01 for FXR-hLBD + OCA vs. FXR-mLBD + OCA; Fig. 6A). Lipoprotein fractionation by FPLC revealed that the reductions of plasma cholesterol upon OCA treatment were due to marked decreases in high-density lipoprotein cholesterol (HDL-C) as well as low-density lipoprotein cholesterol (LDL-C; Fig. 6B).

FXR is a known negative regulator of the HDL biogenesis gene *Apoa1* (44), but in the current study hepatic *Apoa1* expression was similar in all groups (Fig. 6C). Hepatic expression of scavenger receptor class B type I (*Sr-b1*/*Scarb1*), which mediates the selective uptake of HDL-C by the liver (45), was increased 1.4-fold in OCA-treated FXR-mLBD mice and 2.3-fold in FXR-hLBD mice relative to their respective vehicle-treated controls (*P* < 0.01; Fig. 6C), suggesting increased hepatic selective uptake of HDL-derived cholesterol as a likely mechanism for low HDL-C levels in FXR-hLBD mice. The LDL receptor (LDLR) controls circulating LDL-C levels. However, hepatic expression of *Ldlr* and LDLR-degrading proprotein convertase subtilisin/kexin type 9 (*Pcsk9*) was not significantly altered in any of the groups (Fig. 6D). Likewise, expression of *Hmgcr*, encoding the rate-controlling cholesterol synthesis enzyme HMG-CoA reductase, did not appear to be impacted by genotype and OCA treatment (Fig. 6D).

### OCA induces enhanced hepatic injury and cell proliferation signaling in FXR-hLBD mice

**Fig. 7:**
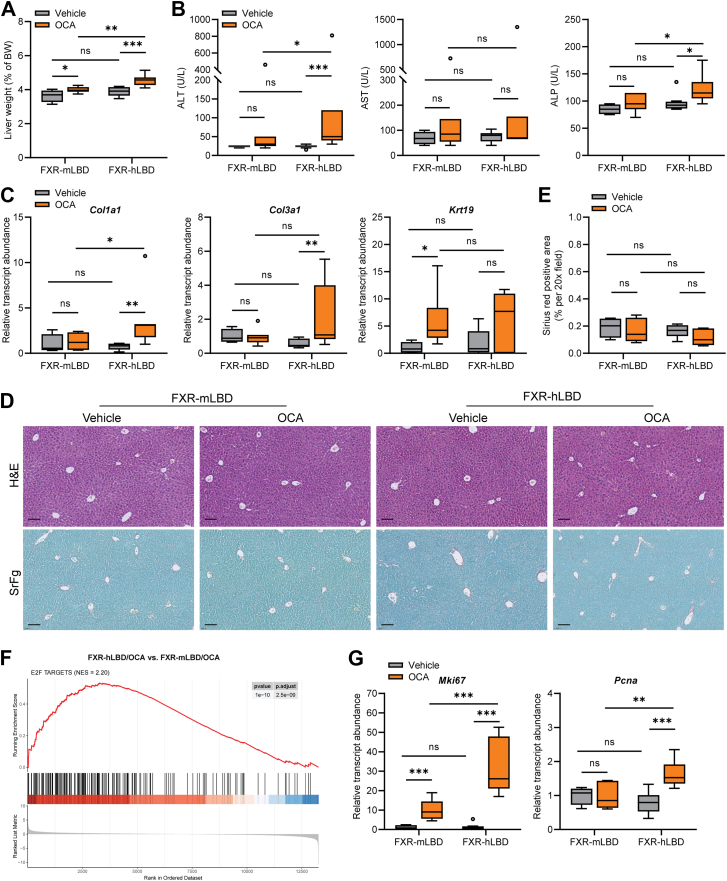
OCA induces markers of hepatic injury in FXR-hLBD mice. (A: Liver weight (% of BW). B: Plasma ALT, AST, and ALP concentrations. C: Hepatic transcript abundance of the fibrogenic genes *Col1a1* and *Col3a1*, and cholangiocyte marker *Krt19*, calculated from normalized RNA-seq data. D: Representative liver sections stained for H&E and SrFg (collagen deposition). Scale bars: 100 μm. E: Quantification of collagen deposition in the livers. (F) GSEA plot of the Hallmark E2F targets gene set, which was significantly upregulated in FXR-hLBD/OCA compared to FXR-mLBD/OCA mice (NES = 2.20; FDR = 2.5 × 10^−9^). G: Hepatic transcript abundance of cell proliferation markers, *Mki67* and *Pcna*, calculated from normalized RNA-seq data. N = 4–8 mice/group. Data are presented as Tukey box-and-whisker plots. ns, not significant, ∗*P* < 0.05, ∗∗*P* < 0.01, ∗∗∗*P* < 0.001 by Kruskal-Wallis H test, followed by Conover post hoc comparisons. ALP, alkaline phosphatase; ALT, alanine aminotransferase; AST, aspartate aminotransferase; BW, body weight; H&E, hematoxylin and eosin; LBD, ligand-binding domain; OCA, obeticholic acid; SrFg, sirus red and fast green.

Finally, we assessed whether OCA and related metabolic changes induced adverse health effects in FXR-hLBD livers. OCA treatment was associated with increased liver weight in both genotypes, with a more pronounced increase in FXR-hLBD mice (+16.7% vs. +7.3%; Fig. 7A). Plasma alanine aminotransferase (ALT) and alkaline phosphatase (ALP) concentrations were elevated in OCA-treated FXR-hLBD mice (Fig. 7B), suggesting liver damage. Consistently, the fibrogenic genes *Col1a1* and *Col3a1* were upregulated by OCA exclusively in FXR-hLBD livers, while the increased expression of the cholangiocyte marker *Krt19* reached statistical significance in FXR-mLBD livers only upon OCA treatment (Fig. 7C). However, these changes in gene expression did not yet translate into clear differences in liver histology in this short-term experiment (Fig. 7D, E). Transcriptomic analysis further revealed that OCA induced upregulation of cell proliferation gene sets, i.e., G2M checkpoint and E2F targets, with more pronounced enrichments in FXR-hLBD than in FXR-mLBD livers (Supplemental Fig. S5 and Fig. 7F). In line with this, expression of proliferation markers *Mki67* and *Pcna* was markedly increased in FXR-hLBD mice compared to FXR-mLBD mice after OCA treatment (Fig. 7G). Taken together, these data indicate that FXR-hLBD mice are more susceptible to OCA-induced hepatotoxicity and display enhanced activation of cell proliferation-related signaling.

## Discussion

We have generated a mouse model with a humanized FXR ligand-binding domain (FXR-hLBD), preserving expression of all 4 FXR isoforms, to enable these mice to respond to (pharmacological) FXR activation in a “human-like” manner. No overt phenotypic differences or alterations in liver transcriptomic profiles were observed between FXR-hLBD and WT (FXR-mLBD) controls of either sex under basal conditions. Also, breeding FXR-hLBD mice into a background with a relatively hydrophobic BA composition, i.e., cross-breeding with *Cyp2c70*-deficient mice (36), did not lead to evident changes in liver function parameters or expression of BA-related genes. Yet, FXR-hLBD mice exhibited clearly enhanced transcriptional and metabolic responsiveness to OCA, highlighting the importance of the species difference in FXR-LBD structure for the outcomes of FXR-targeted pharmacological interventions and, hence, for their translational value.

Since the discovery of BAs as the natural ligands of FXR (46, 47, 48), FXR has been identified as a bona fide target for cholestatic and metabolic liver diseases (10). Several studies have addressed the physiological consequences of treatment with natural and synthetic FXR agonists in healthy mice as well as in mouse models of various liver diseases (10), without taking potential species differences in FXR biology into account. As early as in 1999, Parks *et al.* (46) compared the responses of full-length human and murine FXR upon exposure to various BAs in CV-1 cells, observing trends in differential activation but with large variations. Later, however, Cui *et al.* (20) demonstrated that the coding sequence divergence within the FXR-LBD underlies a major sensitivity difference to CDCA. The substitutions of two amino acid residues, lysine^366^ and valine^384^ (by asparagine and isoleucine, respectively; Supplemental Fig. S2), effectively “humanized” the murine FXR-LBD in vitro (20). However, to the best of our knowledge, no follow-up studies have addressed the physiological and pharmacological consequences of this issue so far. The current study provides the first in vivo evidence that humanization of the murine FXR-LBD confers increased sensitivity to activation by the CDCA analogue OCA in mice. Interestingly, such species-specific sensitivity differences are not unique to FXR. Amino acid variation between human and mouse Takeda G protein-coupled receptor 5 (TGR5/*GPBAR1*) has also been shown to contribute to marked species differences in efficacy of many reported agonists (49, 50).

The presence of isoforms and isoform-specific regulation complicate FXR functions (4, 5, 6, 7). In humans, FXRα1&2 are mostly enriched in the liver, whereas FXRα3&4 predominate in the intestine (3). In mice, all four isoforms are expressed at more or less similar levels in the liver, with FXRα3&4 being most abundant in the intestine (4). FXR-hLBD mice displayed a tissue-specific expression pattern similar to that observed in FXR-mLBD mice (Fig. 2C, D) rather than showing the human isoform distribution pattern, which was anticipated because the endogenous murine *Fxr* promoter was maintained in this model. The expression of all four humanized isoforms suggests that the complex isoform regulation is preserved in FXR-hLBD mice.

Taurine-conjugated MCAs, particularly TαMCA and TβMCA, have been identified as endogenous antagonists in mice and have also been shown to antagonize human FXR in human FXR-based assays (38, 51, 52). Sayin *et al.* demonstrated that TβMCA suppressed TCA-induced *Fgf15* expression in mouse ileal explants. TαMCA and TβMCA antagonize CDCA-induced coactivator recruitment by recombinant human FXR-LBD (38). In line with these findings, Li *et al.* showed that TβMCA inhibited TCA-induced FXR reporter activity in Caco-2 cells transfected with human FXR and human ASBT (51). However, direct quantitative comparisons of MCA antagonist potency against human versus murine FXR-LBD remain limited. In the current study, to our surprise, the presence of FXR-hLBD did not modulate phenotypes or hepatic/ileal expression patterns of BA-related genes in mice with the normal MCA-enriched BA pool under basal conditions, nor when expressed in *Cyp2c70*-deficient mice with a CDCA-enriched BA pool depleted of MCAs (36). This indicates that FXR-controlled processes involved in BA synthesis under these conditions are already highly suppressed. It will be of interest to evaluate regulation of BA synthesis in the context of humanized FXR in situations associated with increased BA production such as feeding with a cholesterol-enriched diet or BA sequestration.

FXR activation is well-known to inhibit BA synthesis through hepatic FXR/SHP and intestinal FXR/FGF15/19 signaling (1). However, previous mouse studies showed that these target genes do not consistently respond to the potent FXR agonist OCA. For example, as summarized by Li *et al.* (25) and as recently reported (53), hepatic *Shp* and/or *Cyp7a1* expression may remain unchanged or show only limited changes in OCA-treated mice compared with their controls, in part depending on dose and mode of administration. In the present study, FXR-hLBD mice showed more pronounced responses to OCA than FXR-mLBD mice, with hepatic *Shp* expression further induced and *Cyp7a1* expression more strongly suppressed. In line with this, ileal *Fgf15* expression was ∼10-fold higher in OCA-treated FXR-hLBD mice relative to vehicle controls, whereas no significant change was observed in FXR-mLBD mice, consistent with previous studies (25, 54, 55). Additionally, OCA significantly induced ileal *Shp* and *Ibabp* expression, indicating that intestinal FXR signaling is activated by OCA in both FXR-mLBD and FXR-hLBD mice (Supplemental Fig. S7). However, the induction of ileal *Shp* was more pronounced in FXR-hLBD mice (∼21-fold vs. vehicle) than in FXR-mLBD mice (∼8-fold vs. vehicle). Intestinal *Ostα* and *Ostβ* were significantly induced by OCA only in FXR-hLBD mice, although their expression levels did not significantly differ between OCA-treated FXR-mLBD and FXR-hLBD mice. Therefore, our data suggest that the responsivity differences between humanized and WT FXR at least partly explain why these earlier studies failed to detect significant transcriptional changes.

Reasonably, we observed that FXR activation more strongly induced hepatic expression of BA efflux transporters and repressed expression of BA uptake transporters in FXR-hLBD livers (1). However, these changes did not lead to elevated plasma BA concentrations. A likely explanation is that all mice were studied under healthy conditions, in which pathological BA accumulation in the liver and spillover into circulation are limited. OCA-induced FXR activation has previously been reported to decrease total BA pool size in WT mice (56). We found that total BA concentrations in the gallbladder bile were decreased in OCA-treated mice, with the lowest concentrations observed in the FXR-hLBD group (Supplemental Fig. S6B), suggesting that total BA pool size was more profoundly reduced in FXR-hLBD mice.

In humans, OCA has been reported to adversely affect plasma lipoprotein profiles in a disease-dependent manner (22, 23, 41, 42, 43). In healthy volunteers, OCA treatment causes increases in plasma cholesterol levels, which are associated with decreased HDL-C (−16%) and increased LDL-C (+22%) concentrations (24, 40), the latter likely inferred by reduced use of cholesterol for BA synthesis. Notably, plasma HDL-C, as a metabolic indicator, is inversely associated with plasma triglyceride levels and risk for ASCVD (16). In contrast to humans, mouse studies have shown that FXR activation reduces plasma total cholesterol, HDL-C, and in some cases LDL-C levels (26, 56, 57). Our study confirms these previous findings and further demonstrates that LBD humanization does not majorly affect overall murine lipoprotein profiles, but does enhance the magnitude of OCA-induced cholesterol changes. The further decrease in HDL-C upon OCA was accompanied by more strongly increased expression of *Sr-b1*, suggesting enhanced reverse cholesterol transport (45). Yet, the mechanism by which OCA contributes to the lowering of LDL-C with simultaneous suppression of BA synthesis in mice remains to be elucidated.

We found that OCA promoted mild liver injury in healthy FXR-hLBD mice but not in FXR-mLBD controls, even though liver damage was not histologically apparent in this short-term experiment. Similarly, hepatotoxic effects of OCA (e.g., liver fibrosis and elevated liver enzymes) have been reported to be mediated through FXR activation in bile duct ligation and MASLD mouse models (58, 59). These findings suggest the enhanced FXR activation in FXR-hLBD mice may sensitize the liver to agonist-induced liver injury. The absence of clear histological abnormalities in our study indicates that these changes represent limited early responses. In addition, we found that OCA treatment, especially in FXR-hLBD mice, led to increased liver weight, consistent with previous findings studied in healthy and cholestatic animals (58, 60) and concomitant upregulation of cell proliferation-related genes. In this context, it is interesting to note that Huang *et al.* revealed that BA buildup by CA feeding in WT mice induced FXR activation, thereby not only suppressing BA synthesis but also stimulating DNA synthesis and liver growth to manage the BA overload (61).

OCA received conditional approval by the FDA and EMA in 2016 for PBC treatment based on results of the POISE trial (22). Although recent real-world data reported beneficial effects of OCA in PBC patients (62, 63), its clinical use is most notably limited by the dose-dependent worsening of pruritus (64). In addition, concerns have been raised about the potential for OCA-related liver injury in both cirrhotic and non-cirrhotic PBC patients (65). The insufficient demonstration of clinical efficacy in the phase 4 COBALT trial (NCT02308111), combined with the safety concerns, led to early termination of the COBALT trial and revocation of OCA’s European marketing authorization in late 2024 . OCA was also voluntarily withdrawn from the US market in September 2025. Overall, our findings suggest that the presence of FXR-hLBD may sensitize the liver to OCA-associated liver injury, underscoring the potential value of our model for the preclinical assessment of FXR agonists. Recently, a new steroidal selective FXR agonist, TC-100/INT-787, has been developed as a water-soluble derivative of OCA, with reduced detergent properties and potentially less toxicity (66). Hence, TC-100 may represent a promising alternative to OCA for the treatment of cholestatic liver diseases. Future studies are needed to evaluate the therapeutic effects of TC-100 and other emerging FXR modulators. The use of humanized mouse models, such as the FXR-hLBD model, represents a novel strategy that may improve the translational relevance of preclinical assessments of FXR-targeting compounds.

In summary, we developed a mouse model with a humanized FXR by substituting the murine LBD with the human LBD, preserving all four FXR isoforms and their tissue-specific expression patterns. FXR-hLBD mice exhibit a normal phenotype under basal conditions, even in the context of a CDCA-enriched BA pool but do show enhanced transcriptional and metabolic responsiveness to OCA administration compared with WT (FXR-mLBD) controls. Taken together, these findings indicate that FXR-hLBD mice represent a valuable model for evaluating FXR-targeted (pharmacological) interventions in a translation-relevant manner.

## Data availability

All data generated or analyzed during this study are included in this published article and its supplementary information files.

## Supplemental data

This article contains supplemental data (20).

## Conflict of interest

The authors declare the following financial interests/personal relationships which may be considered as potential competing interests: Henkjan J. Verkade is consultant of Albireo/Ipsen, Intercept, Mirum, Orphalan, ProQR and Vertex. Jan Freark de Boer and Folkert Kuipers are consultants for Dexoligo Therapeutics. All consultancy fee payments are made to the University Medical Center Groningen. All other authors disclose no conflicts of interest.
