# Supplementary material for: Mice with humanized FXR ligand-binding domain display distinct metabolic responses upon pharmacological FXR stimulation
Source: J Lipid Res. 2026 Jun 25;67(8):101088. doi: 10.1016/j.jlr.2026.101088 (PMC13396712; doi:10.1016/j.jlr.2026.101088)
Supplement: Supplementary Material [file mmc6.docx]

**Supplementary Materials:**

**Mice with humanized FXR ligand-binding domain display distinct metabolic responses upon pharmacological FXR stimulation**

Jinxiao Li^1^, Hilde D. de Vries^1^, Kirill Ustyantsev^2^, Milaine V. Hovingh^1^, Niels L. Mulder^1^, Rick Havinga^1^, Nicolette Huijkman^1^, Sarah Falcone^1^, Krisztina de Bruyn^3^, Ellen Weersing^2^, Niels J. Kloosterhuis^1^, Marieke Smit^1^, Eugene Berezikov^2^, Bart van de Sluis^1^, Henkjan J. Verkade^1^, Folkert Kuipers^2,3,#,*^, Jan Freark de Boer^1,3,#,*^

**Affiliations**

^1^Department of Pediatrics, University of Groningen, University Medical Center Groningen (UMCG), Groningen, The Netherlands.

^2^European Research Institute for the Biology of Ageing (ERIBA), University of Groningen, University Medical Center Groningen (UMCG), Groningen, The Netherlands.

^3^Department of Laboratory Medicine, University of Groningen, University Medical Center Groningen (UMCG), Groningen, The Netherlands.

^#^ These authors equally contributed to this study

^*^ Correspondence


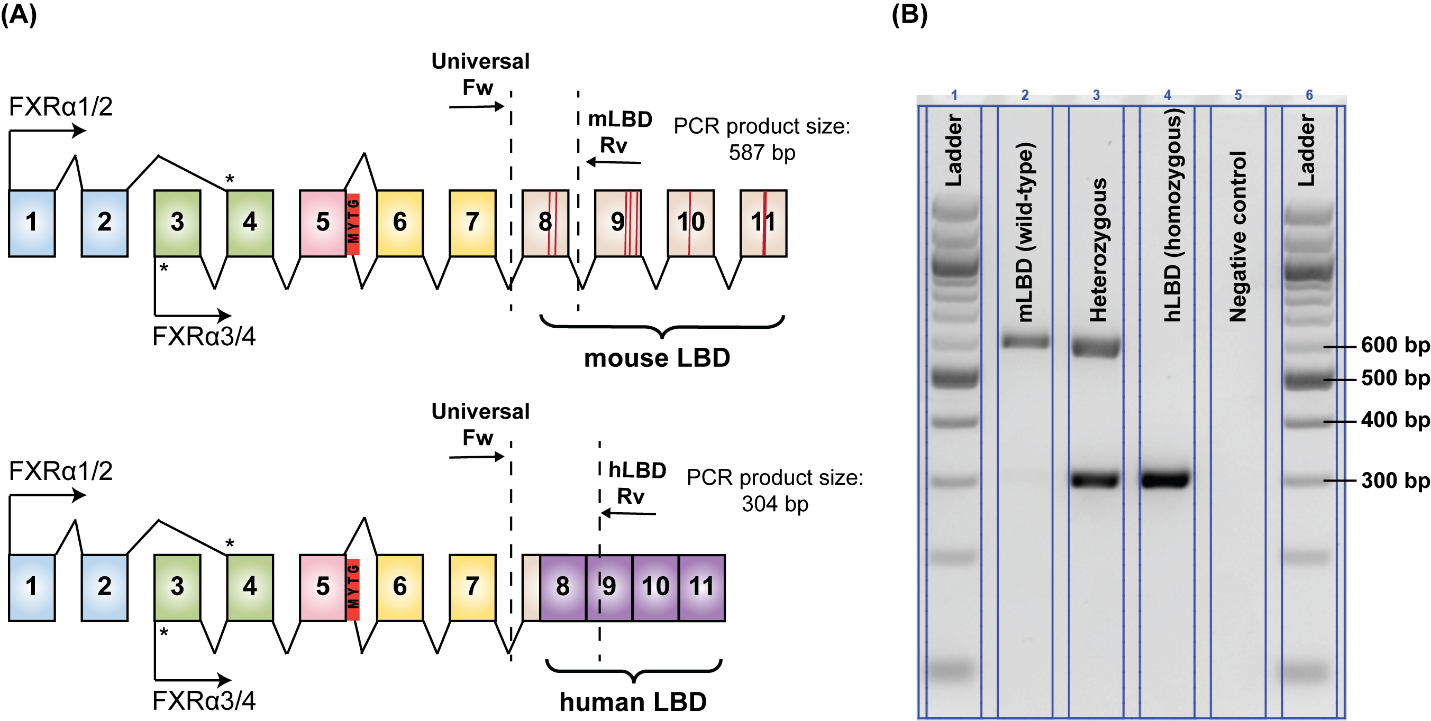


**Supplementary Figure S1. Genotyping strategy for FXR-hLBD mice.**

(A) Schematic representation of primer positions for genotyping. The universal forward primer (Fw) was combined with either a reverse primer (Rv) specific to the murine ligand-binding domain (LBD) or a reverse primer specific to the human LBD. (B) Representative PCR products of FXR-mLBD (wild-type; 587 bp), heterozygous (587 bp and 304 bp), and FXR-hLBD (homozygous; 304 bp) mice, as well as a negative control on a 2% agarose gel. A 100 bp DNA ladder was used.


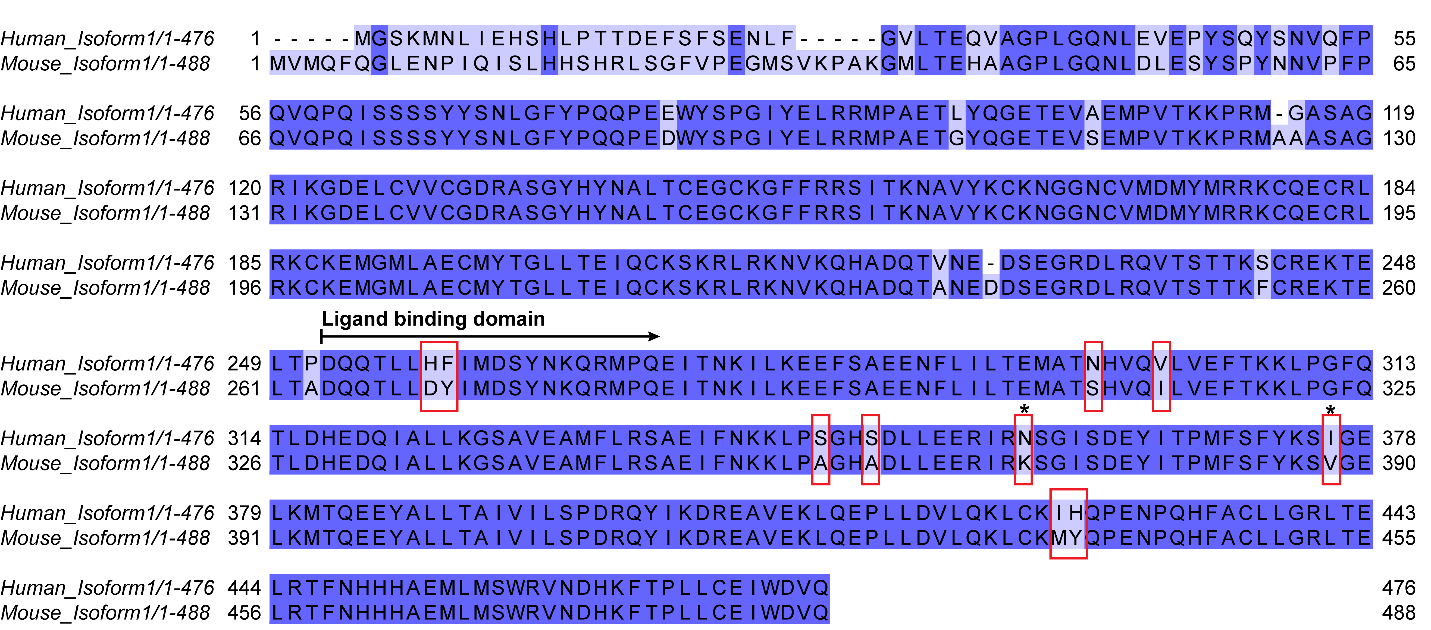


**Supplementary Figure S2. Amino acid sequence alignment of human and mouse FXR.** The start position of FXR ligand-binding domain is indicated. The accession numbers used for human and mouse FXR are NP_001193908 and NP_001157172, respectively. Amino acid differences in the ligand-binding domain between the two receptors are highlighted with red boxes. Asterisks indicate replacements of lysine^366^ (K) and valine^384^ (V) in mouse FXR, which has been reported to modulate receptor sensitivity differences between mice and humans *in vitro* [1].


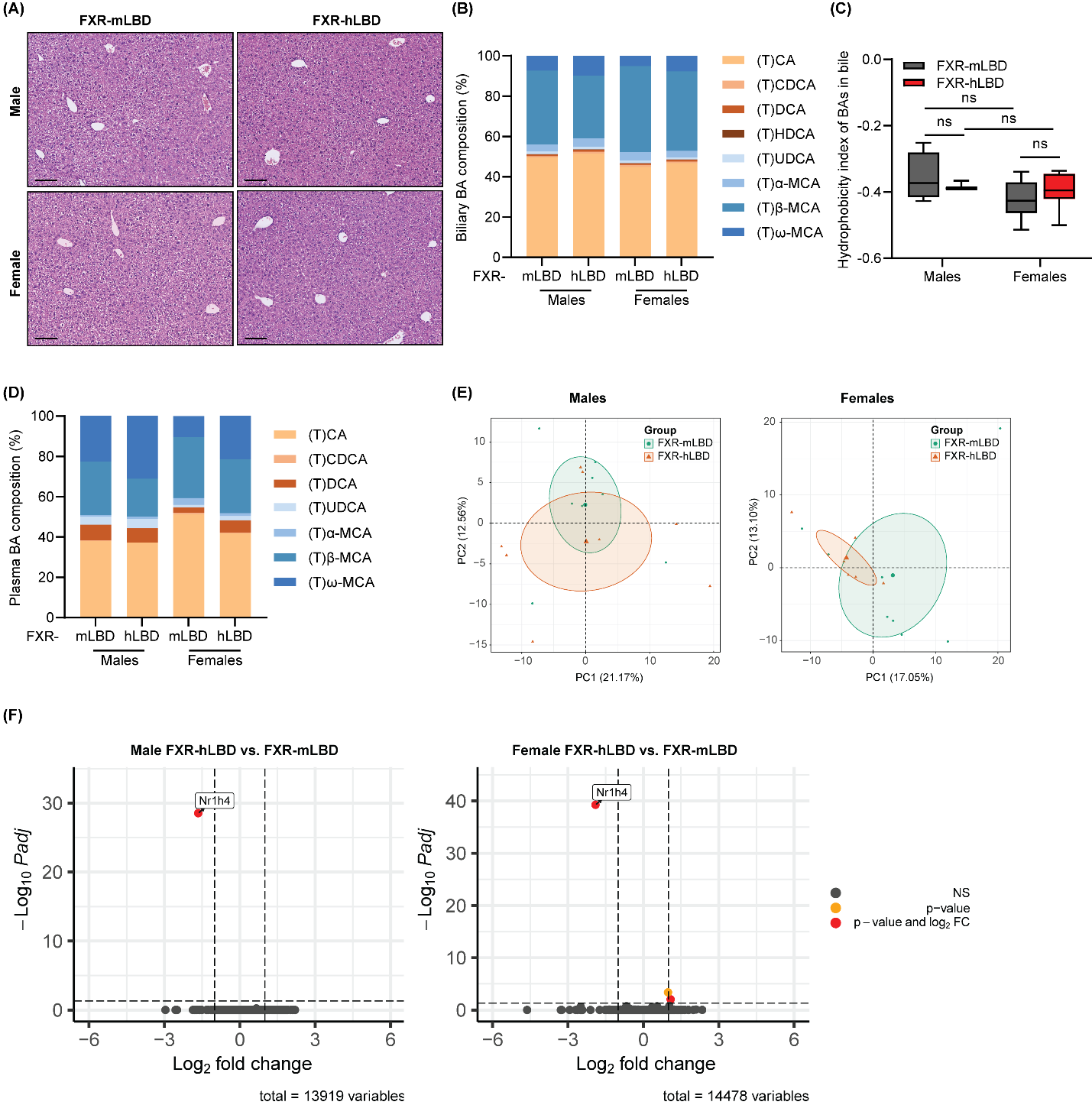


**Supplementary Figure S3. Bile acid composition and hepatic transcriptome profiles are similar between FXR-hLBD and FXR-mLBD mice under basal conditions.**

(A) Representative H&E staining of liver sections from FXR-mLBD and FXR-hLBD mice. Scale bars: 100 µm. (B) Biliary BA composition. (C) Hydrophobicity index of BAs in gallbladder bile. ns, not significant by Kruskal-Wallis H test, followed by Conover post hoc comparisons. (D) Plasma BA composition. (E) PCA of liver RNA-seq data from male (left) and female (right) FXR-mLBD and FXR-hLBD mice. Each dot represents a biological replicate. (F) Volcano plots showing differentially expressed genes in FXR-hLBD vs. FXR-mLBD livers from males (left) and females (right). *Nr1h4* (*Fxr*) was detected as differentially expressed due to the mapping bias, as reads from the human LBD sequence align less efficiently to the mouse reference genome. N = 7-8 mice/group. BA, bile acid; LBD, ligand-binding domain; PC, principal component.

**
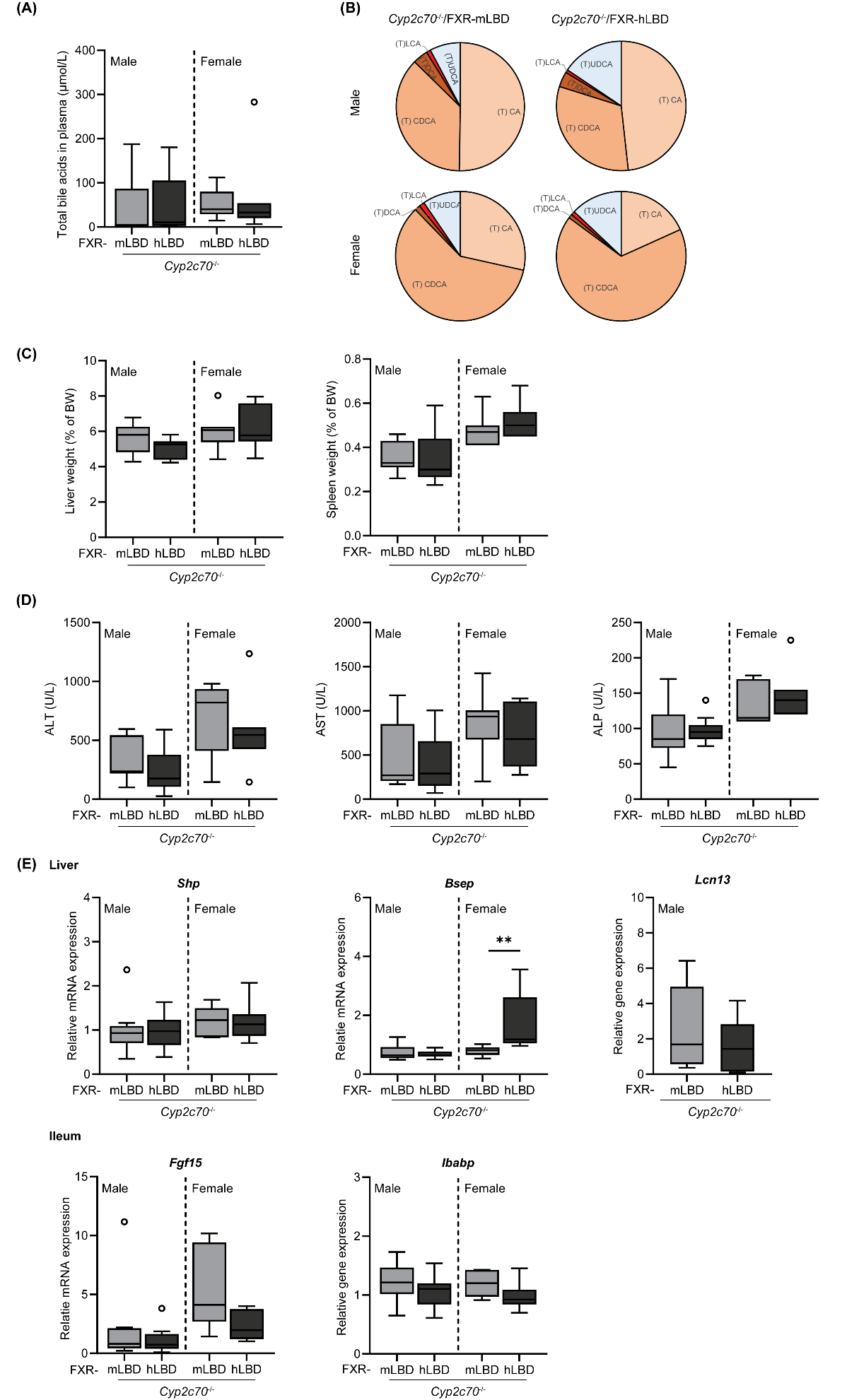
**

**Supplementary Figure S4. FXR-LBD humanization does not induce phenotypic changes in mice with a context of human-like bile acid composition under basal conditions.**

(A) Total plasma BA concentrations. (B) Plasma BA composition. (C) Liver and spleen weights (% of BW). (D) Plasma ALT, AST, and ALP concentrations. (E) Relative mRNA expressions of *Shp*, *Bsep*, and *Lcn13* (liver), as well as of *Fgf15* and *Ibabp* (ileum), quantified by RT-qPCR. N = 7-13 mice/group. ***P* < 0.01 by Mann-Whitney U test. ALP, alkaline phosphatase; ALT, alanine aminotransferase; AST, aspartate aminotransferase; BA, bile acid; BW, body weight; LBD, ligand-binding domain.

**
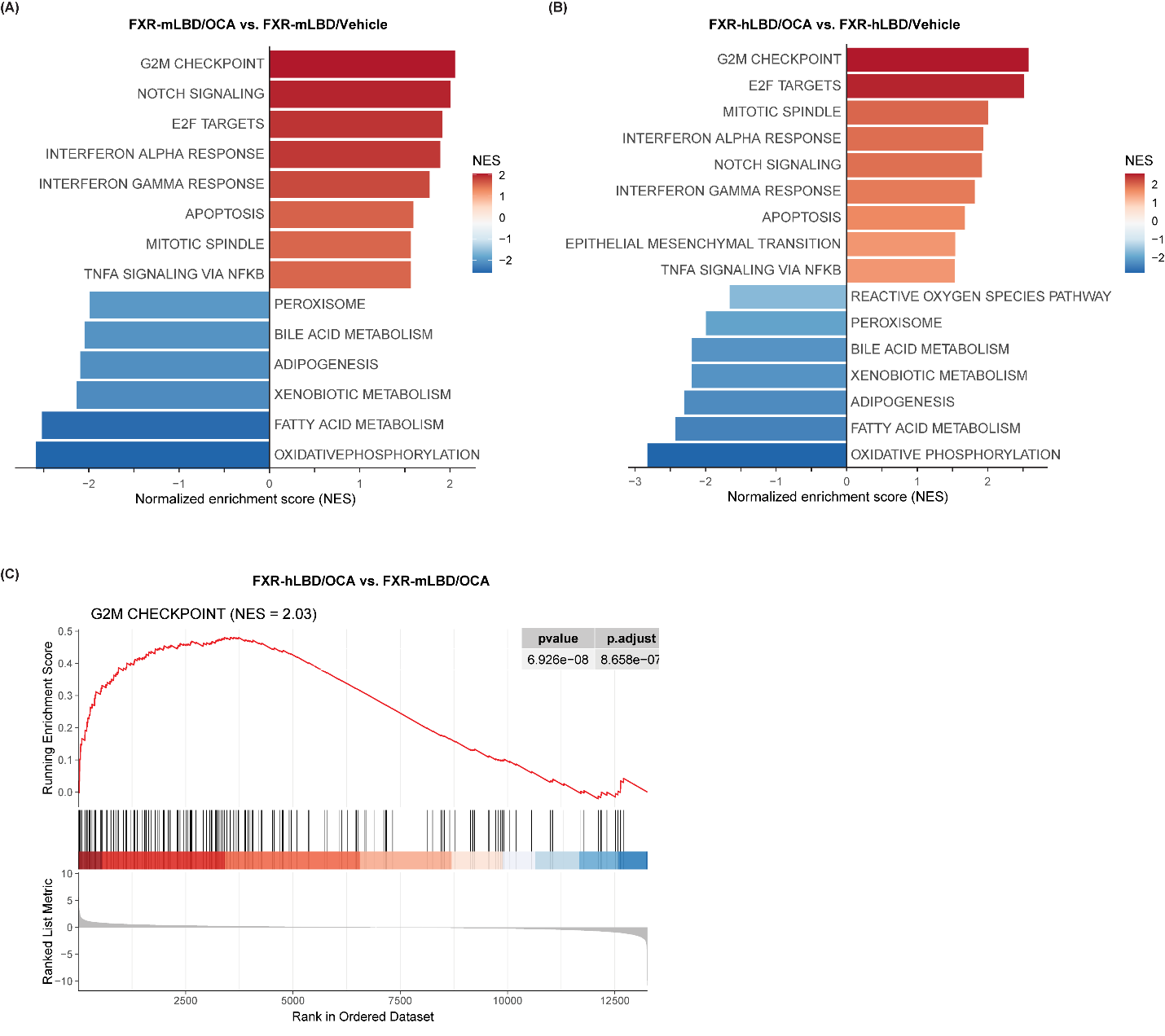
**

**Supplementary Figure S5. Gene set enrichment analysis using Hallmark gene sets in FXR-hLBD and FXR-mLBD livers upon OCA treatment.**

(A-B) Top upregulated and downregulated Hallmark gene sets in FXR-mLBD/OCA versus FXR-mLBD/vehicle mice (A) and FXR-hLBD/OCA versus FXR-hLBD/vehicle mice (B) (FDR < 0.05). DESeq2 output was ranked by the Wald statistic, prior to GSEA. (C) Hallmark G2M checkpoint gene set in FXR-hLBD/OCA versus FXR-mLBD/OCA mice. LBD, ligand-binding domain; OCA, obeticholic acid.

**
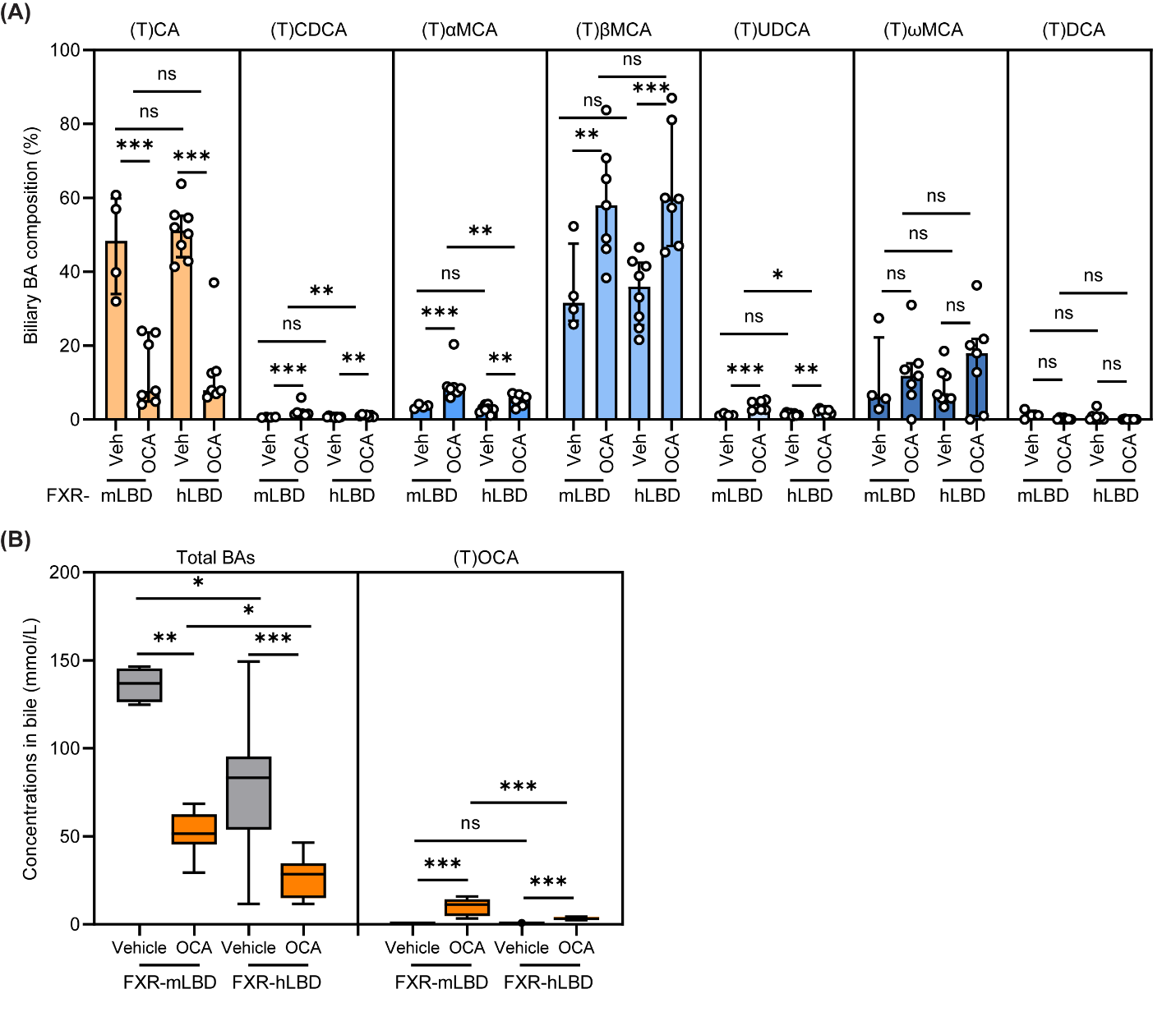
**

**Supplementary Figure S6. Bile acid composition and concentrations in gallbladder bile.** (A) Biliary bile acid composition. Data are presented as a bar plot showing median with interquartile range. (B) Total bile acid and (T)OCA concentrations in bile. N = 4-8 mice/group. Data are presented as Tukey box-and-whisker plots. ns, not significant, **P* < 0.05, ***P* < 0.01, ****P* < 0.001 by Kruskal-Wallis H test, followed by Conover post hoc comparisons. BA, bile acid; LBD, ligand-binding domain; OCA, obeticholic acid.

**
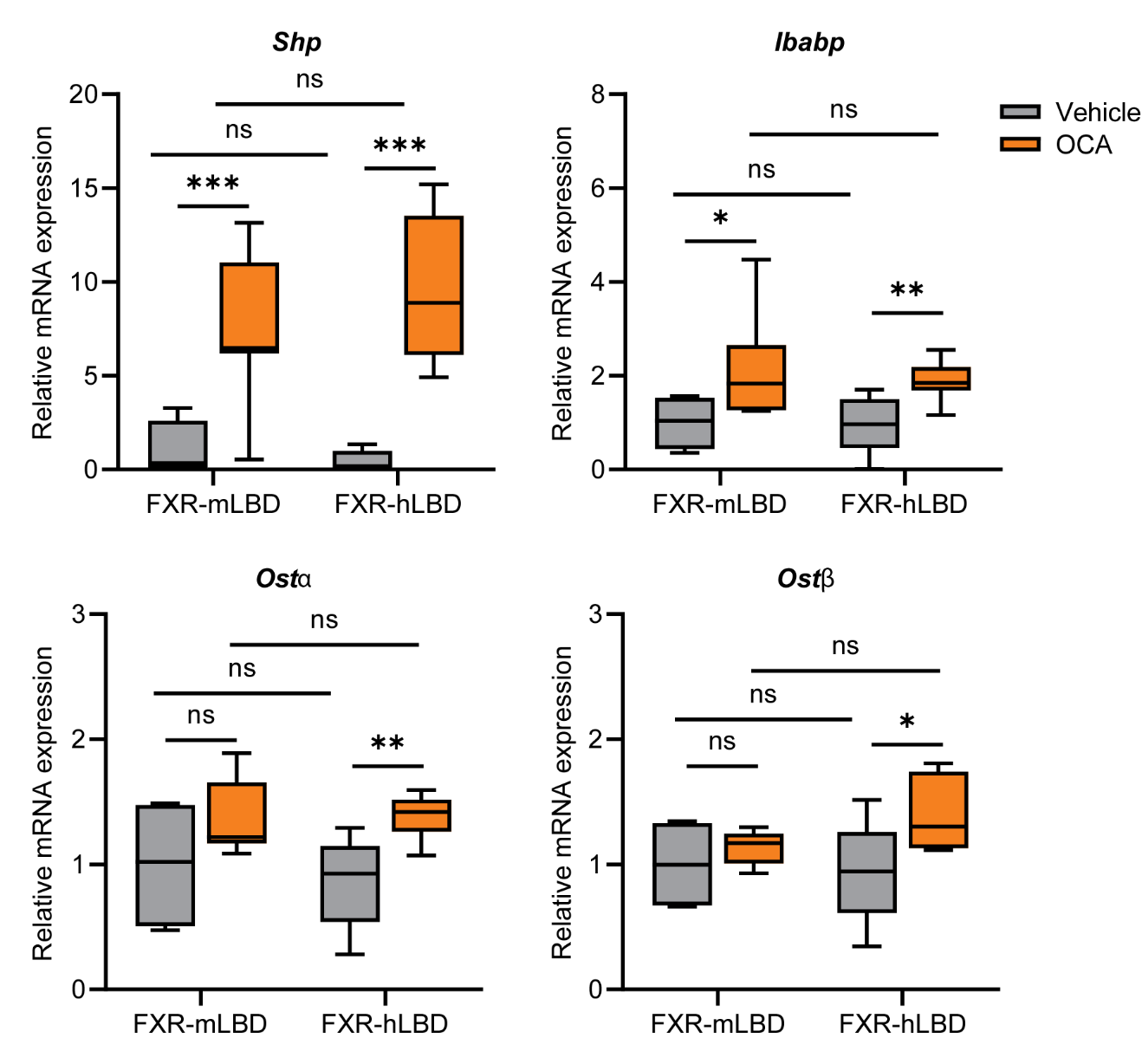
**

**Supplementary Figure S7. Ileal mRNA expression of the intestinal *Fxr*-target genes, quantified by RT-qPCR.** Data are presented as Tukey box-and-whisker plots. ns, not significant, **P* < 0.05, ***P* < 0.01, ****P* < 0.001 by Kruskal-Wallis H test, followed by Conover post hoc comparisons. LBD, ligand-binding domain; OCA, obeticholic acid.


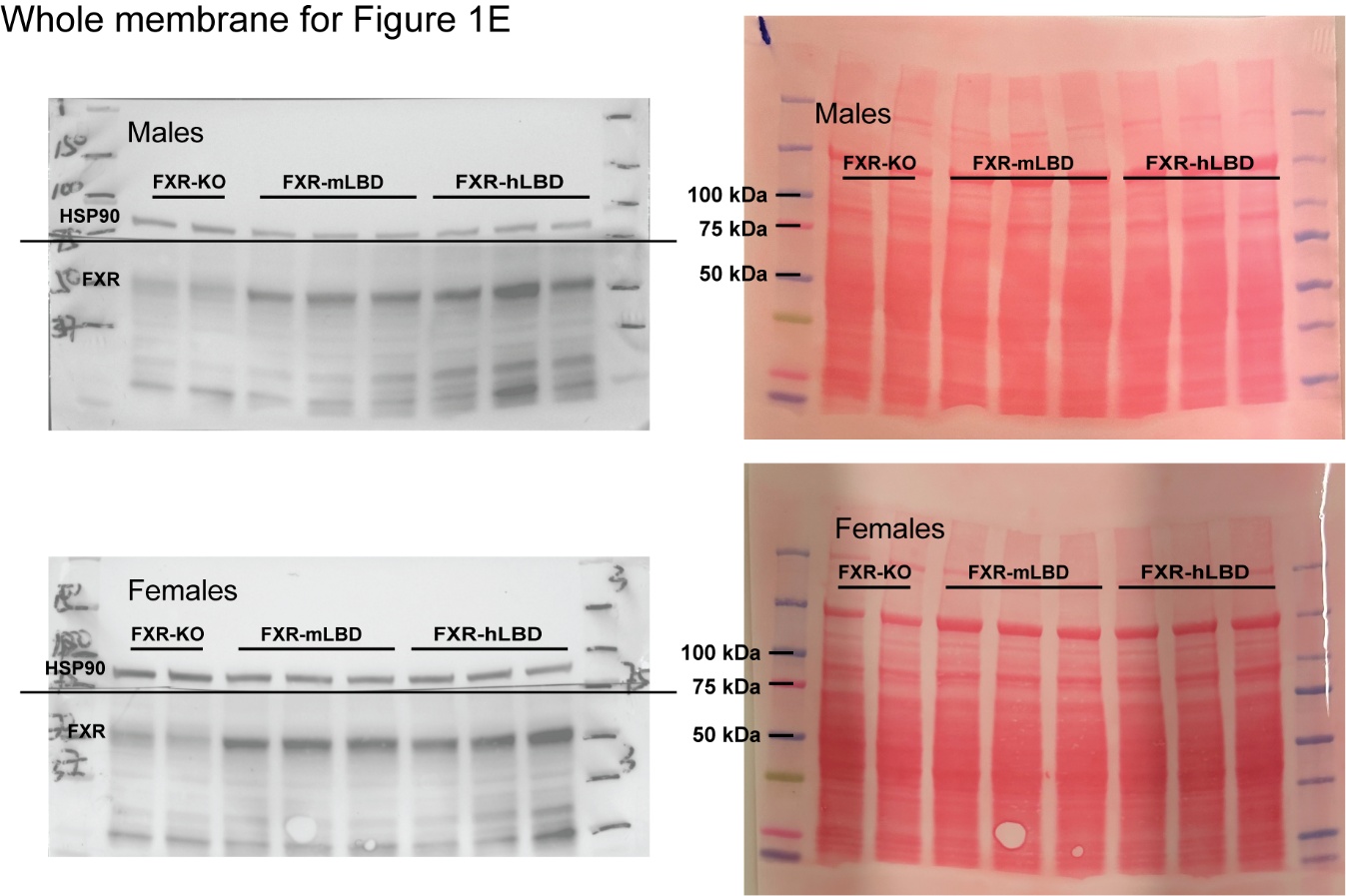


**Supplementary Figure S8. Whole membrane western blot images.**

Original western blot images and Ponceau Staining for Figure 1E. Membranes were cut at approximately 75 kDa prior to antibody incubation to allow separate blotting for HSP90 and FXR proteins. The cutting position is indicated by a black line on the blot.

**Supplementary Table S1.** The DNA repair template sequence used in this study. The homology arms are shown in lowercase.

| DNA Repair template sequence | ttgtaaatggtaatggttttccaaatatgacctgtgtcttaaaaattcattattcaagtcaggtttttgtcctctaacaattttatgt  tgggttattcttttcctgtctatttattaattatttactttttcttaaaatttagTTAAAAGAAGAATTTAGTGCAGAAG  AAAATTTTCTCATATTAACAGAAATGGCAACCAATCAcGTtCAGGTTCTTGTAGAATTC  ACAAAAAAGCTACCAGGATTTCAGACTTTGGACCATGAAGACCAGATTGCTTTGCT  GAAAGGGTCTGCGGTTGAAGCTATGTTCCTTCGTTCAGCTGAGATTTTCAATAAGA  AACTTCCGTCTGGGCATTCTGACCTATTGGAAGAAAGAATTCGAAATAGTGGTATCT  CTGATGAATATATAACACCTATGTTTAGTTTTTATAAAAGTATTGGGGAACTGAAAATG  ACTCAAGAGGAGTATGCTCTGCTTACAGCAATTGTTATCCTGTCTCCAGATAGACAA  TACATAAAGGATAGAGAGGCAGTAGAGAAGCTTCAGGAGCCACTTCTTGATGTGCT  ACAAAAGTTGTGTAAGATTCACCAGCCTGAAAATCCTCAACACTTTGCCTGTCTCCT  GGGTCGCCTGACTGAATTACGGACATTCAATCATCACCACGCTGAGATGCTGATGT  CATGGAGAGTAAACGACCACAAGTTTACCCCACTTCTCTGTGAAATCTGGGACGTG  CAGTGATGGGCCATGGCCCAACTTGTTTATTGCAGCTTATAATGGTTACAAATAAAG  CAATAGCATCACAAATTTCACAAATAAAGCATTTTTTTCACTGCATTCTAGTTGTGGTT  TGTCCAAACTCATCAATGTATCTTATCATGTCTGGATCTCAGTCATGTACAGATTCTCG  TAGAATTCACAAAAAAGCTTCCAGgtattttttttaaataataaaaattaatgttcttgaaatatgtaaagtgtgc  Cattataatattaatcactctgtatatgatattttatttcagttcttaaagcattctttttgtttgttttcaagacagggtttctctgtgtagc  cctggctgtcc |
| --- | --- |

**Supplementary Table S2.** Primer sequences used in this study.

| Gene | Species | Sequence | |
| --- | --- | --- | --- |
| *Fxr*  (Genotyping) | Mus musculus | forward | 5’-AGGAAGTCTCAGGTCTATCTTGT-3’ (universal) |
|  |  | reverse | 5’-GAAAAACACCTAGCAAAAGAAATCC-3’ (WT/mLBD allele) |
|  |  | reverse | 5’-TCAGCAAAGCAATCTGGTCTT-3’ (hLBD allele) |
| *Cyclophilin G* | Mus musculus | forward | 5’-GGGGATAAAGGTCCAGCGT-3’ |
|  |  | reverse | 5’-AGACAACTCTTCCAGCAGGT-3’ |
| *Fxr-mLBD* | Mus musculus | forward | 5’-TGGATTCGTACAACAAACAGAGA-3’ (universal) |
|  |  | reverse | 5’-TCTGTACATGACTGGTTGCCA-3’ |
| *Fxr-hLBD* | Homo sapiens | forward | 5’-TGGATTCGTACAACAAACAGAGA-3’ (universal) |
|  |  | reverse | 5’-TGTGAATTCTACAAGAACCTGAACG-3’ |
| *Fxra1&2* | Mus musculus | forward | 5’-CTTTCTGAA AGCTTATTTGGTATGCTA A-3’ |
|  |  | reverse | 5’-AGTACGATTCCAAATCCAGATTCTG-3’ |
| *Fxra3&4* | Mus musculus | forward | 5’-GTGAAGCCAGCTAAAGGTATGCTAA-3’ |
|  |  | reverse | 5’-AGTACGATTCCAAATCCAGATTCTG-3’ |
| *Fxra1&3* | Mus musculus | forward | 5’-GAGGGCTGCAAAGGTTTCTTC-3’ |
|  |  | reverse | 5’-AGTTAACAAACCTGTATACATACATTCAGC-3’ |
| *Fxra2&4* | Mus musculus | forward | 5’-GAGGGCTGCAAAGGTTTCTTC-3’ |
|  |  | reverse | 5’-TGGATTTCAGTTAACAAACATTCAGC-3’ |
| *Shp* | Mus musculus | forward | 5’-CACGATCCTCTTCAACCCAGATGT-3’ |
|  |  | reverse | 5’-CCAGGGCTCCAAGACTTCAC-3’ |
| *Bsep* | Mus musculus | forward | 5’-GACTTTCCACAGTGGCGTCT-3’ |
|  |  | reverse | 5’-TCACTCAACAACCCTACAGATG-3’ |
| *Lcn13* | Mus musculus | forward | 5’-GGAAGCCCCGCCAGAT-3’ |
|  |  | reverse | 5’-TGGCCTTTGCGTACCAGATC-3’ |
| *Ostα* | Mus musculus | forward | 5’-TTTGCTCTGTTCCAGGTGCTT-3’ |
|  |  | reverse | 5’-TAGGGAGGTGAGCAAGCGAT-3’ |
| *Ostβ* | Mus musculus | forward | 5’-GAGCATCCTGGCAAACAGAAAT-3’ |
|  |  | reverse | 5’-GGGGCCAAGTCTGGTTTCTC-3’ |
| *Ibabp* | Mus musculus | forward | 5’-GGCAAGAAGTTCAAGGCTACC-3’ |
|  |  | reverse | 5’-CGCTCATAGGTCACATCCCC-3’ |
| *Fgf15* | Mus musculus | forward | 5’-GACTGCGAGGAGGACCAAAA-3’ |
|  |  | reverse | 5’-ACGTCCTTGATGGCAATCGT-3’ |

**References**

[1] Cui, J., Heard, T.S., Yu, J., Lo, J.-L., Huang, L., Li, Y., et al., 2002. The Amino Acid Residues Asparagine 354 and Isoleucine 372 of Human Farnesoid X Receptor Confer the Receptor with High Sensitivity to Chenodeoxycholate. Journal of Biological Chemistry 277(29): 25963–9, Doi: 10.1074/jbc.M200824200.
